# Dietary zinc supplementation inhibits bacterial plasmid conjugation *in vitro* by regulating plasmid replication (*rep*) and transfer (*tra*) genes

**DOI:** 10.1128/aem.01480-24

**Published:** 2024-10-03

**Authors:** Logan Ott, Chloe Smith, Melha Mellata

**Affiliations:** 1 Interdepartmental Microbiology Graduate Program, Iowa State University, Ames, Iowa, USA; 2 Department of Food Science and Human Nutrition, Iowa State University, Ames, Iowa, USA; Universita degli Studi di Napoli Federico II, Portici, Italy

**Keywords:** diet, zinc, zinc gluconate, horizontal gene transfer (HGT), multidrug-resistant (MDR), plasmid conjugation

## Abstract

Humans use dietary supplements for several intended effects, such as supplementing malnutrition. While these compounds have been developed for host end benefits, their ancillary impact on the gut microbiota remains unclear. The human gut has been proposed as a reservoir for the prevalent lateral transfer of antimicrobial resistance and virulence genes in bacteria through plasmid conjugation. Here, we studied the effect of dietary zinc supplements on the incidence of plasmid conjugation *in vitro*. Supplement effects were analyzed through standardized broth conjugation assays. The avian pathogenic *Escherichia coli* (APEC) strain APEC-O2-211 was a donor of the multidrug resistance plasmid pAPEC-O2-211A-ColV, and the human commensal isolate *E. coli* HS-4 was the plasmid-free recipient. Bacterial strains were standardized and mixed 1:1 and supplemented 1:10 with water, or zinc derived from either commercial zinc supplements or zinc gluconate reagent at varying concentrations. We observed a significant reduction in donors, recipients, and transconjugant populations in conjugations supplemented with zinc, with a dose-dependent relationship. Additionally, we observed a significant reduction (*P* < 0.05) in log conjugation efficiency in zinc-treated reactions. Upregulation of the mRNA for the plasmid replication initiation gene *repA* and the subset of transfer genes *M*, *J*, *E*, *K*, *B*, *P*, *C*, *W*, *U*, *N*, *F*, *Q*, *D*, *I*, and *X* was observed. Furthermore, we observed a downregulation of the conjugal propilin gene *traA* and the entry exclusion gene *traS*. This study demonstrates the effect of dietary zinc supplements on the conjugal transfer of a multidrug resistance plasmid between pathogenic and commensal bacteria during *in vitro* conditions.

**IMPORTANCE:**

This study identifies dietary zinc supplementation as a potential novel intervention for mitigating the emergence of multidrug resistance in bacteria, thus preventing antibiotic treatment failure and death in patients and animals. Further studies are required to determine the applicability of this approach in an *in vivo* model.

## INTRODUCTION

The emergence of multidrug-resistant (MDR) bacteria is driven by the lateral spread of antimicrobial resistance (AMR) through horizontal gene transfer (HGT) and is a global health concern (1–4). The process of HGT is primarily driven by the direct lateral exchange of large AMR plasmids between bacteria (5–7). The gut of humans and other animals is proposed as a potent reservoir for this lateral exchange of plasmids between typically naive bacterial populations and transient pathogens or pathobionts (8–10). Commensal bacteria may serve as a microbial reservoir for MDR plasmids in the gut or may begin to express AMR and virulence determinants, leading to the emergence of novel pathogens (8–11). Innovative approaches are urgently needed to prevent this acquisition and spread of resistance genes to mitigate global AMR concerns (12).

Dietary supplements target numerous desired physiological outcomes, including weight loss, muscle function, alertness, sleep, digestion, and supplementation of essential vitamins and minerals (13). These compounds often have supporting literature for their intended and marketed uses; however, little is typically understood about their interaction with the host gut microbiota. Additionally, many factors can affect the rate and incidence of bacterial plasmid conjugation in the gut of animals; however, much of the interaction between bacteria and the host is not yet well characterized (5, 14–16). We previously demonstrated that when all other factors are held constant, the host genetics alone can regulate bacterial conjugation in the gut of a defined microbiota murine model (17). Others have demonstrated that dietary components, such as medium-chain fatty acids, inhibit bacterial conjugation *in vitro* (18, 19). As such, many host factors may have unanticipated roles in regulating bacterial interactions in the gut.

For example, zinc has been shown to have robust antimicrobial activity and is frequently studied as a preservation ingredient for food and cosmetic products (20, 21). While the exact mechanism of zinc antimicrobial action is unclear, the accepted mechanism is the dissociation of zinc derivatives into charged zinc ions (Zn^2+^) in the gut; charged zinc ions then readily react with cellular components to form reactive oxygen species (ROS). Generated ROS further interact with extra- and intracellular molecules, which leads to cell membrane damage, leakiness, and reduction and damage to sugars, proteins, and nucleic acids (20–22).

However, how dietary supplements affect the ability of bacteria to participate in successful plasmid conjugation has yet to be examined. This study aims to evaluate the effect of dietary zinc supplements on the incidence of bacterial plasmid conjugation in a minimal *in vitro* model for the animal gut. We identified inhibitory effects of supplementation on donor, recipient, and transconjugant populations, as well as a reduction in the frequency of conjugation of the MDR plasmid pAPEC-O2-211A-ColV between the avian pathogenic *Escherichia coli* (APEC) strain APEC-O2-211 and the human commensal gut *E. coli* strain HS-4. At last, we identified the regulatory role of zinc exposure on the expression of replication (*rep*) and transfer (*tra*) gene expression.

## MATERIALS AND METHODS

### Chemicals and reagents

Zinc tablets (Spring Valley, Walmart, Bentonville, AR, USA) were purchased from a local supermarket. Zinc tablets were crushed via mortar and pestle and suspended in sterile double-distilled (dd)H_2_O at a final zinc concentration of 10 mg/mL. Active ingredients were allowed to dissolve into solution for 30 minutes and cleared of the residue by centrifugation at 900 × *g* for 15 minutes. The supernatant was transferred to a new sterile conical tube through passage through a 0.22-µM syringe filter. Sterile filtrates were then stored at 4°C until used in conjugation assays. Zinc gluconate was obtained from VWR (Cat. # Z1077-500GM; VWR, Radnor, PA, USA) and dissolved in ddH_2_O at the same final concentrations as zinc tablets. To enumerate zinc ions in supplement and reagent derived zinc gluconate solutions, a colorimetric zinc assay kit (Cat. # ab102507; Abcam, Cambridge, United Kingdom) was used according to the manufacturer’s instructions. The absorbance of kit reactions was determined using an automated plate spectrophotometer (SpectraMax M2e, Molecular Devices, San Jose, CA) and measured at 560 nm. The included zinc standard was used to generate a standard curve for which the concentrations of unknown samples were interpolated.

### Bacterial strains and growth

The bacterial plasmid donor for all assays was the APEC-O2-211 strain that contains one large conjugative MDR plasmid pAPEC-O2-211A-ColV (NZ_CP030791.1) and two smaller non-mobile accessory plasmids pAPEC-O2-211B (NZ_CP030792.1) and pAPEC-O2-211C (NZ_CP030793.1). The pAPEC-O2-211A-ColV plasmid is a hybrid IncFIB/IncFIC plasmid that confers resistance to macrolides, nitroimidazole, aminocoumarin, fluoroquinolone, tetracycline, carbapenem, and aminoglycoside antibiotics (23). APEC-O2-211 is a substrain of APEC-O2, a zoonotic pathogen that results in colibacillosis, an extraintestinal infection in chickens, and demonstrates potential as a gastrointestinal pathogen in humans (4, 11, 23, 24). The plasmid-free human gut commensal bacterial strain *E. coli* HS-4, used as the recipient for all conjugation assays in this study, is a derivative of the *E. coli* HS strain with chromosomal resistance to rifampicin (17). Strains were maintained as cryogenic stocks at −80°C and streaked out on fresh selective media before each experiment.

Strains were plated on MacConkey agar supplemented with either tetracycline (donor, 15 µg/mL) or rifampicin (recipient, 100 µg/mL) for isolation and selection for purity. MacConkey agar was also used to differentiate between donor and recipient cells by lactose fermentation. APEC-O2-211 is lactose positive and HS-4 is lactose negative, allowing for the differentiation between donor and recipients and spontaneous rifampicin-resistant donors. Before conjugation assays, cultures were grown overnight in Luria-Bertani (LB, Miller) broth supplemented with 0.1% glucose and appropriate antibiotic under shaking conditions (225 rotations per minute) at 37°C to exponential phase to an OD_600 nm_ of >1.0 (17, 25).

### *In vitro* broth conjugation assays

To measure the effect of zinc supplementation on conjugation, standard *in vitro* broth conjugation was conducted with some modifications (17, 25). Briefly, overnight donor and recipient broth cultures were standardized to an OD_600 nm_ of ~1.0 via centrifugal concentration and phosphate-buffered saline rinsed thrice. The rinsed, standardized pellets were resuspended to their final desired volume with fresh antibiotic-free LB broth supplemented with 0.1% v/v sterile glucose. Donor and recipient strains were mixed at a 1:1 ratio and immediately supplemented 1:10 with either ddH_2_O or zinc solution in individual wells of a 96-well cell culture plate. The conjugation mixtures were then pipet homogenized to ensure complete mixing of donors, recipients, and the compound of interest and then incubated at 37°C for 3 hours in aerobic conditions to prevent the contribution of transconjugant replication and transconjugant donation of the recipient plasmids. Following incubation, reactions were serially diluted in 10-fold increments and plated on MacConkey agar supplemented with tetracycline (donor, 15 µg/mL), rifampicin (recipient, 100 µg/mL), or tetracycline and rifampicin (transconjugants, 15 and 100 µg/mL, respectively) (17, 25). In order to avoid bias due to variations in donor or recipient populations, the logarithm of conjugation efficiency was calculated as


(Eq. 1) $$Log\;conjugation\;efficiency={log}_{10}\left(\frac{T}{\sqrt{D\times R}}\right)$$

where *T* is the total CFU per milliliter of transconjugants; *D* is the total CFU per milliliter of donors; and *R* is the total CFU per milliliter of recipients (26).

### Quantitative RT-qPCR assays for detecting changes in plasmid replication and transfer gene expression

For primer design, the complete list of coding sequences (CDS) for pAPEC-O2-211A-ColV (NZ_CP030791.1) was obtained from National Center for Biotechnology Information (NCBI), and the CDS for *rep* and *tra* genes (Table 1) were selected and submitted to the NCBI Primer Blast tool for primer pair production (27). The default settings were used with an exemption for amplicon length, where 75–200 bp were used as search criteria. Furthermore, specificity was checked against the non-redundant nucleotide sequence database with the requirement “bacteria (taxid:2).” All sequences with 100% sequence homology to the gene template were selected as intended targets. Primers were tested via traditional PCR to amplify target genes and specificity.

**TABLE 1:** Primers used for quantitative PCR in this study^*a*^^*,^b^*^

Gene	Primer	Sequence	Amplicon (bp)	*T*_m_ (°C)
Housekeeping				
*16S*	F R	5′-ATACCGCATAACGTCGCAAGACCA-3′ 5′-AGCCGTTACCTCACCAAGCTA-3′	94	64.7 61.2
Replication				
*repA*	F R	5′-GCAGAGCTGGGACTGATTAC-3′ 5′-CATCAAGGGCGGCAAATAAC-3′	106	58.1 57.8
*repB*	F R	5′-AGCTGACACGTCTTTCCCTG-3′ 5′-GGCGGGCAAAGGAATGAATG-3′	123	60.0 60.2
Transfer				
*traA*	F R	5′-GGCGGGCAAAGGAATGAATG-3′ 5′-TGAAGTTCTGGTCGGTGCAG-3′	101	60.2 60.2
*traB*	F R	5′-AGCCGAGGATTTTGCCGTTA-3′ 5′-ATCAGTTGCCTGAAGGACGG-3′	118	60.0 60.0
*traC*	F R	5′-CAGCTGGTTACGGTAGGTGG-3′ 5′-TGACCTGCCTGCCCTTTATG-3′	167	60.1 60.0
*traD*	F R	5′-ACCACCATATCACCACGCTG-3′ 5′-TGGCGATCTGCCGATTATCC-3′	129	60.1 60.0
*traE*	F R	5′-AAACCAGAGTGCCCCAACAA-3′ 5′-GCCTCGGAGATCTGTGCTTT-3′	75	60.0 60.1
*traF*	F R	5′-GCCTCGGAGATCTGTGCTTT-3′ 5′-GCAGGCTGGCAGTGGTATAA-3′	159	60.2 60.1
*traG*	F R	5′-TAACTGTCGCCAATCAGCGT-3′ 5′-ATGCCCAGCAGCTATTTGGT-3′	70	60.0 60.0
*traH*	F R	5′-CTGCGGGTCAACCAGGTATT-3′ 5′-ACACGCCTCTGGATGACAAG-3′	80	60.0 60.0
*traI*	F R	5′-AACCCCGTTTTCCCCACTTT-3′ 5′-CTGGCTTCCACACGGGTTAT-3′	197	60.0 60.0
*traJ*	F R	5′-GTGCGCTGGATCGTAGAGAA-3′ 5′-ACAAACCGGAACGGGAGAAT-3′	101	59.9 59.6
*traK*	F R	5′-ATCTGCCACTGAAGACAGCC-3′ 5′-AACGTCCACGCCTTACGAAT-3′	139	60.0 60.0
*traL*	F R	5′-CCAGAACCGCTGCACCAATA-3′ 5′-AACACTGACCAACCAGAGCC-3′	125	60.7 60.2
*traM*	F R	5′-GCGTCATATACACGGAGCCC-3′ 5′-TGAAAAGCGTCGTCAGGAGG-3′	96	60.7 60.3
*traN*	F R	5′-TCCGGATTCGCGTTATAGCC-3′ 5′-AGATCAAAGGGCAGGGAAGC-3′	79	60.0 60.0
*traP*	F R	5′-TTCATCTGCGGTTACCACCC-3′ 5′-GCATTATGGGCAACACTGGC-3′	111	60.0 60.2
*traQ*	F R	5′-CATGCATCCAGCACCTGGTA-3′ 5′-CATATCGTCGCCCGTCTTGT-3′	110	60.1 60.2
*traR*	F R	5′-ACAACGTCACACCGGGAAAT-3′ 5′-AGTGATGAAGCCGATGAAGCA-3′	169	60.2 60.1
*traS*	F R	5′-TCTGCCAGCAAACGAAGAAATG-3′ 5′-CAGGTCATAAAGCCAGGGGG-3′	74	60.0 60.1
*traT*	F R	5′-TTGCGATTGATTTGGCCAGC-3′ 5′-CAACGGATAATGTTGCCGCC-3′	180	60.1 60.2
*traU*	F R	5′-CTGCAACGGACTGGACTCAT-3′ 5′-GCCAGCGCCTTTAATATGCC-3′	108	60.0 60.0
*traV*	F R	5′-TGCTCACCTGTTCTTAGCGG-3′ 5′-CAGAAACTGCTGGCACCTCT-3′	116	60.0 60.3
*traW*	F R	5′-ATTTTCTGCCCTCCTCTGCC-3′ 5′-CAGAAGTCTCTGGACAGCCG-3′	151	60.0 60.1
*traX*	F R	5′-CCGACCAGAAACATCCACGA-3′ 5′-CATCAAAACGGTGGCACTGG-3′	87	60.0 60.0
*traY*	F R	5′-TTCGGCCACCTCCCTGAATA-3′ 5′-GGCGGTATAAGCAGAACGGT-3′	177	60.6 60.2

^*a*^ *F*, forward; R, reverse; *T*_m_, theoretical melting temperature as calculated via Primer3.

^*b*^ Coding sequences from pAPEC-O2-211A-ColV (NZ_CP030791.1) were used as the template for primer design.

For the reverse transcription-quantitative PCR (RT-qPCR) reactions, cell pellets of *in vitro* conjugations with and without zinc supplementation were harvested at 3 hours of incubation by centrifugation and stored at −80°C until processing. Conjugation reactions used in RNA extraction and RT-qPCR were conducted in triplicate, and each triplicate was measured in duplicate. Pellets were thawed by the addition of TRIzol reagent, and total RNA was extracted following the manufacturer’s instructions (Invitrogen, Waltham, MA, USA). Genomic DNA was removed from RNA by RNase-free DNase I treatment following manufacturer recommendations (Cat. # EN0521; Thermo Fisher, Waltham, MA, USA). RNA purity and concentration were evaluated using a NanoDrop Lite spectrophotometer and Qubit four fluorometer, respectively (Thermo Fisher). According to the manufacturer’s instructions, DNA-free RNA was then converted to cDNA via a high-capacity cDNA reverse transcription kit (Cat. # 4368813, Thermo Fisher).

The resulting cDNA was used as the template for quantitative PCR (qPCR) on a QuantStudio 3 using the PowerTrack SYBR Green qPCR Master Mix according to manufacturer instructions (Cat. # A46109; Applied Biosystems, Waltham, MA, USA). The two-step “fast” qPCR cycle was used, consisting of an initial denaturation phase (20 s, 95°C), 40 cycles of amplification phase (1 s, 95°C; 20 s, 60°C), and a continuous melt phase (1 s, 95°C; 20 s, 60°C; 1 s, 95°C with a ramp speed of 0.1°C/s). Fluorescence was measured during the combined annealing and extension phase for amplification and the temperature ramp in the melt curve phase. The qPCR primers used in this study are described in Table 1. The geometric mean of the 16S rRNA reference gene for each sample was used as the reference index. The 2^−ΔΔCT^ method for relative gene expression was used, and statistics were conducted on the log-transformed 2^−ΔΔCT^ values to normalize sample distribution and prevent skewness (28).

### Statistical analysis

All statistics were performed using the GraphPad Prism version 6 software suite according to the manufacturer’s instructions (GraphPad Software, Boston, MA, USA). Differences between ddH_2_O-treated and supplement-treated reactions were determined via multiple *t*-test analysis. For dose-response assays; differences between the ddH_2_O treatments and individual concentrations were determined via one-way analysis of variance and post hoc Dunnett’s correction for multiple comparisons. For this study, *P* values of ≤0.05 were considered significant.

## RESULTS

### Supplement-derived zinc gluconate has antimicrobial and conjugation inhibition effects

**Fig 1:**
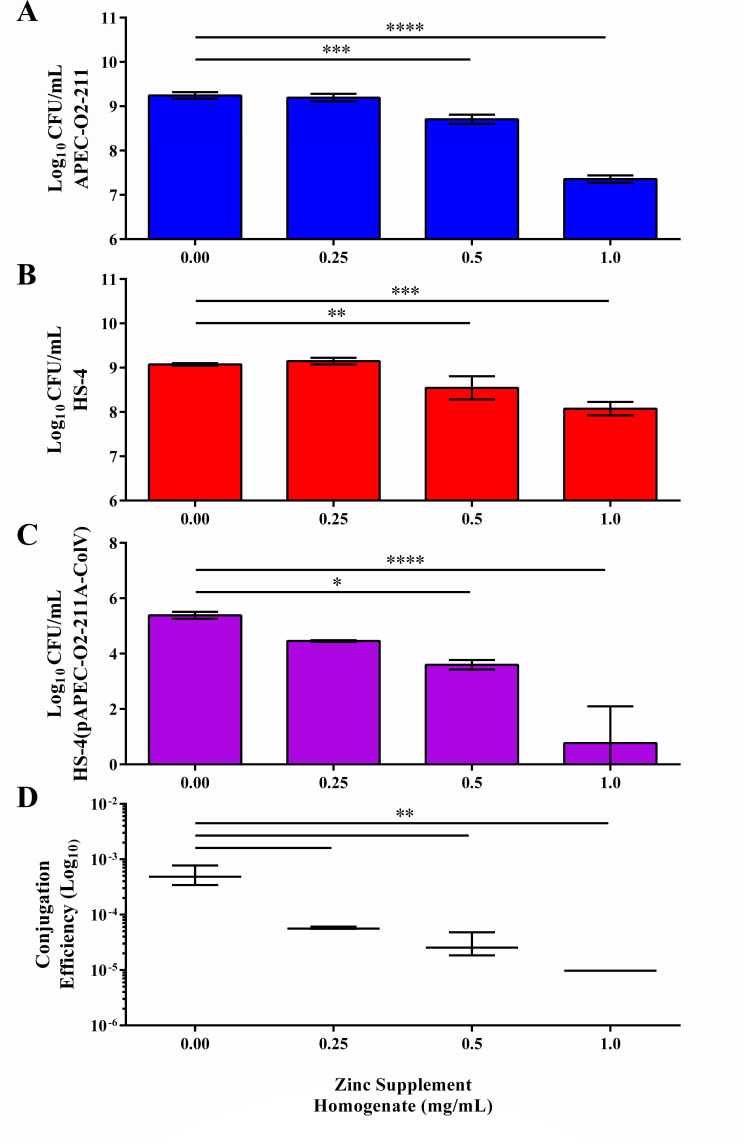
Effect of dietary zinc gluconate tablets on bacterial conjugation *in vitro*. Donors (A, blue), recipients (B, red), transconjugants (C, purple), and conjugation efficiency calculated as described in Equation 1 (**D**) of conjugations supplemented with dietary zinc supplement homogenate at 0-, 0.25-, 0.50-, and 1.00-mg/mL final concentrations. Bars represent the means of triplicate values, and lines represent the standard deviation around the means (**A–C**) or the median and standard errors (**D**). *P < 0.05, **P < 0.005, ***P < 0.0005, ****P < 0.00005.

To determine the effect of supplement-derived zinc on the incidence of bacterial plasmid conjugation, *in vitro* broth conjugations were conducted using the *E. coli* APEC-O2-211 donor strain and the *E. coli* HS-4 recipient strain (Fig. 1). A significant reduction (*P <* 0.05) in the donor strain population CFU per milliliter was observed in conjugations with either 0.50 (0.54 ± 0.10 log) or 1.00 (1.89 ± 0.09 log) mg/mL supplement-derived zinc (Fig. 1A). A significant reduction (*P <* 0.05) in the recipient strain population CFU per milliliter was observed in conjugations with either 0.50 (0.53 ± 0.26 log) or 1.00 (1.00 ± 0.15 log) mg/mL supplement-derived zinc (Fig. 1B). A significant reduction in the transconjugant strain population CFU per milliliter was observed in reactions supplemented with 0.50 (1.78 ± 0.17 log, *P <* 0.05) and 1.00 (4.62 ± 1.33 log, *P <* 0.00005) mg/mL of zinc (Fig. 1C). Reductions in donor, recipient, and transconjugant population CFU per milliliter demonstrated a supplement-derived zinc dose-dependent effect (Fig. 1A through C).

To determine if conjugation efficiency was affected outside of the observed bactericidal effect, log conjugation frequencies were calculated for each reaction as described in Equation 1 (Fig. 1D). Significant (*P <* 0.0005) dose-dependent reduction in mean log conjugation efficiency was detected in 0.25 (10.37-fold), 0.50 (16.93-fold), and 1.00 (431.78-fold) mg/mL of supplement-derived zinc-treated conjugations compared to the ddH_2_O-treated control (Fig. 1D).

### Reagent-derived zinc gluconate demonstrates a dose-dependent inhibitive and antimicrobial response

**Fig 2:**
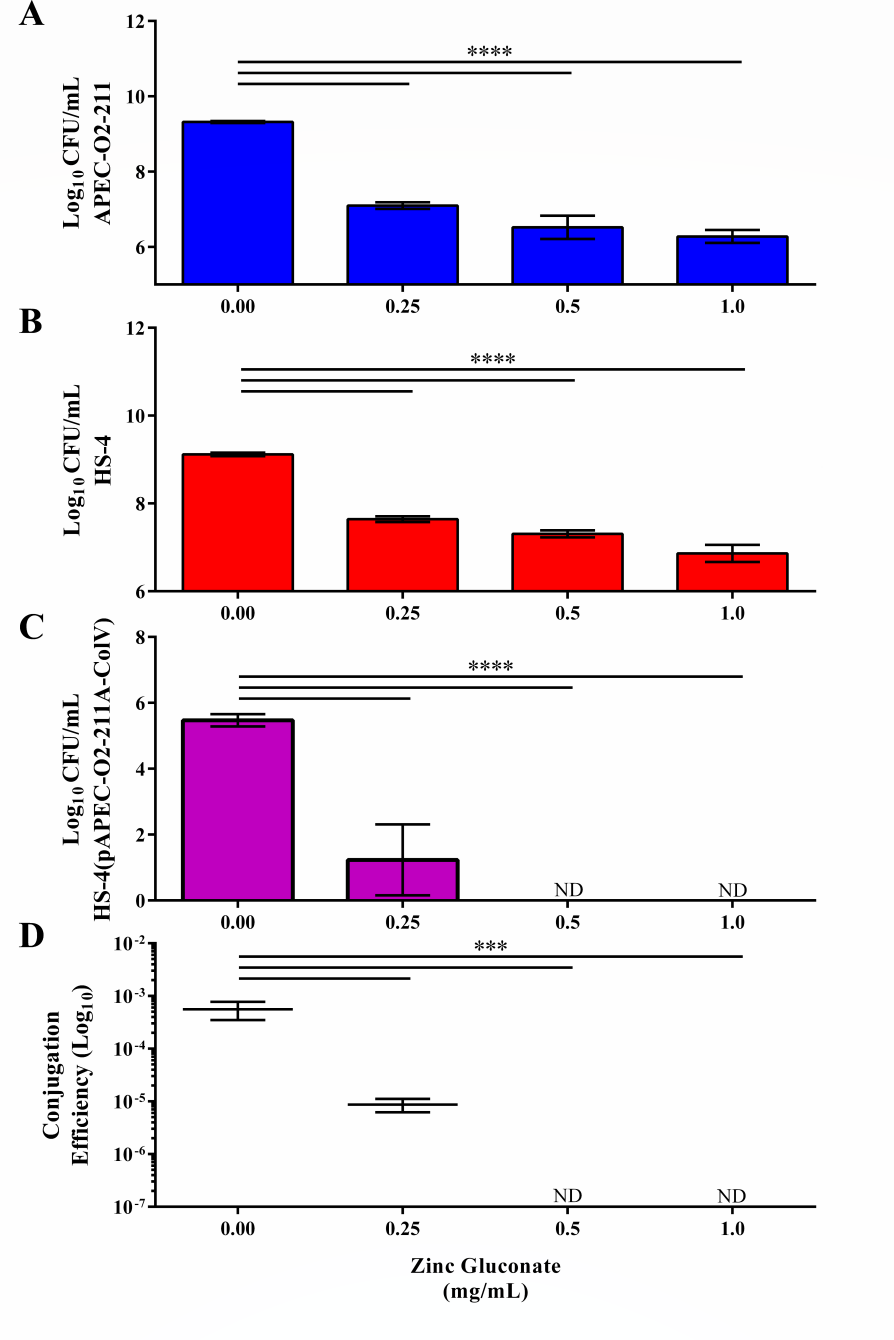
Effect of pure zinc gluconate on bacterial conjugation *in vitro*. Donors (A, blue), recipients (B, red), transconjugants (C, purple), and conjugation efficiency calculated as described in Equation 1 (**D**) of conjugations supplemented with dietary zinc supplement homogenate at 0-, 0.25-, 0.50-, and 1.00-mg/mL final concentrations. Bars represent the mean of triplicate values, and lines represent the standard deviation around the means (**A–C**) or the median and standard errors (**D**). ***P < 0.0005, ****P < 0.00005. ND, not detected.

To determine if zinc was the active species mediating reduction in donor, recipient, and transconjugant CFU per milliliter in supplement-derived zinc solutions, pure zinc gluconate was tested at equivalent concentrations (Fig. 2). A significant decrease (*P <* 0.00005) in the donor strain population CFU per milliliter was observed in conjugations supplemented with 0.25 (2.22 ± 0.09 log), 0.50 (2.80 ± 0.31 log), or 1.00 (3.04 ± 0.17 log) mg/mL of reagent-derived zinc (Fig. 2A). A significant reduction (*P <* 0.00005) in the recipient strain population CFU per milliliter was observed in conjugations supplemented with 0.25 (−1.48 ± 0.06 log), 0.50 (−1.81 ± 0.08 log), or 1.00 (−2.25 ± 0.20 log) mg/mL of reagent-derived zinc (Fig. 2B). A significant reduction in the transconjugant strain population CFU per milliliter was observed in reactions supplemented with 0.25 (−4.24 ± 1.08 log, *P <* 0.0005) mg/mL of reagent-derived zinc (Fig. 2C). Furthermore, transconjugant CFUs were not detected in reactions supplemented with 0.50 or 1.00 mg/mL of reagent-derived zinc (Fig. 2C).

Log conjugation efficiencies were calculated for each reaction as described in Equation 1 to determine if log conjugation efficiency was affected outside of the observed bactericidal effect of reagent-derived zinc (Fig. 2D). A significant (*P <* 0.0005) reduction in mean log conjugation efficiency was detected in 0.25 (219.64-fold) mg/mL of zinc-treated conjugations compared to the ddH_2_O-treated control (Fig. 2D). Log conjugation efficiencies were not calculable for 0.50- and 1.00-mg/mL treatments as no transconjugants were detected in these treatment groups.

**Fig 3:**
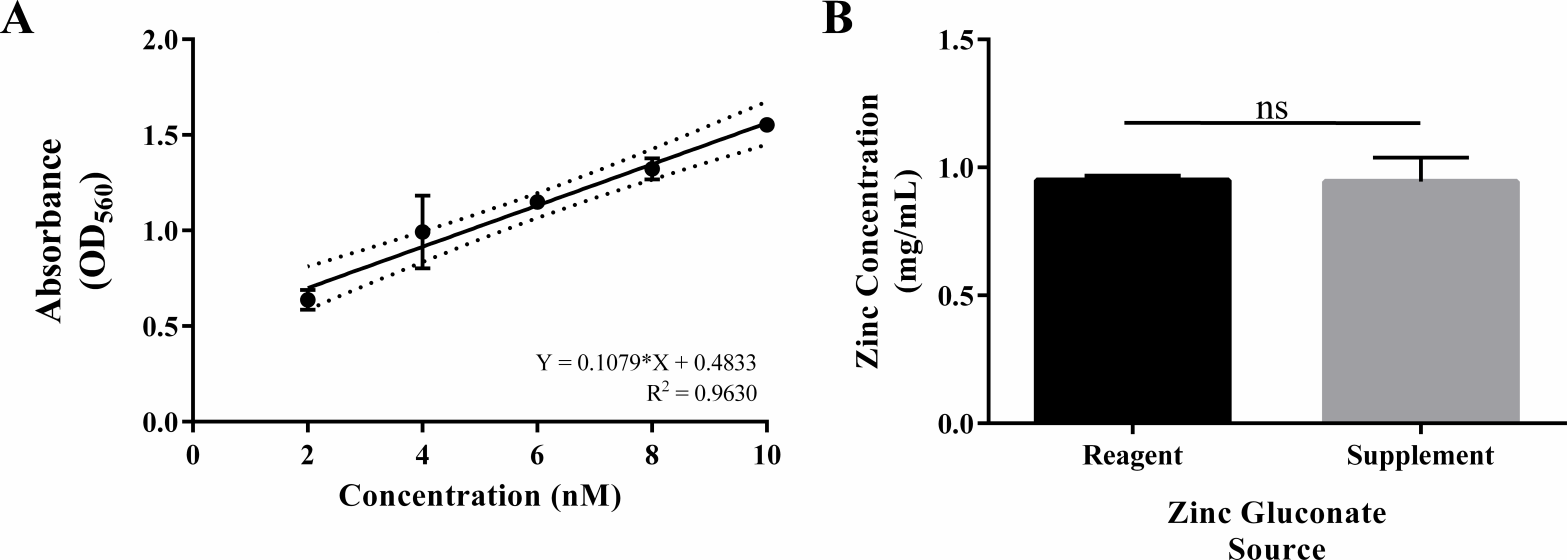
Zinc ion quantitation from supplement- and reagent-derived zinc gluconate solutions. (**A**) Linear regression of zinc assay standard curve showing line of best fit (solid) with correlated 95% confidence intervals (dashes). (**B**) Unknown sample enumeration via interpolation of sample absorbance from the standard curve line of best fit. Bars represent the mean of three replicates, and lines represent the standard error around the means. ns, not significant.

To determine if differences observed between supplement- and reagent-derived zinc gluconate were due to variations in zinc ion concentration in prepared test solutions, zinc ions were enumerated using a zinc assay kit. No significant differences in zinc ion concentration were observed between supplement- and reagent-derived zinc gluconate solutions (Fig. 3).

### Reagent-derived zinc supplementation affects the expression of plasmid replication and transfer genes

**Fig 4:**
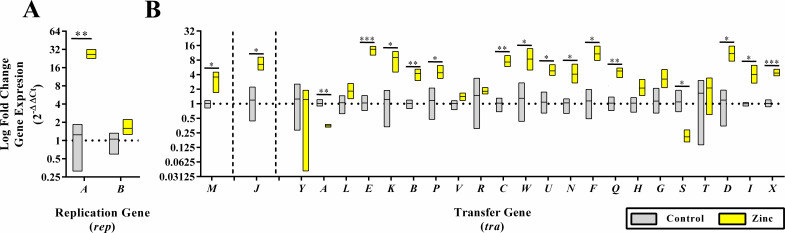
RT-qPCR of plasmid replication and conjugation gene expression during *in vitro* zinc gluconate supplemented conjugation reactions. Log fold changes in gene expression for replication (*rep*, A) and transfer (*tra*, B) genes under control (gray) and zinc (yellow) supplementation. Genes presented in the order observed on the plasmid sequence. Vertical dashed lines represent the location of known promoters. Bars represent the arithmetic mean of triplicate reactions, each evaluated in duplicate. Error bars represent the sample standard error. Log fold change in gene expression was calculated via the 2^−ΔΔCt^ method. *P* values below or equal to 0.05 were considered significant. *P < 0.05, **P < 0.005, ***P < 0.0005.

To elucidate the mechanism for zinc-associated plasmid conjugation inhibition, we measured gene expression relating to the replication (*rep*) and transfer (*tra*) of plasmids. RT-qPCR was conducted to measure the expression of plasmidic replication regulator and integral conjugation genes (Table 1; Fig. 4). The mRNA for the replication initiator protein RepA was significantly (*P <* 0.005) upregulated (26.43 ± 4.56-fold) in conjugations supplemented with 1.00 mg/mL of zinc compared to ddH_2_O-treated controls (Fig. 4A). The mRNA for the protein RepB showed a numerical increase (1.58 ± 0.53-fold) but was not significant (*P* = 0.243) (Fig. 4A). Furthermore, the mRNAs for the conjugal pilus protein TraA and the surface exclusion protein TraS were significantly (*P <* 0.005) downregulated (2.88 ± 0.18-fold and 5.16 ± 1.46-fold, respectively) in conjugations supplemented with 1.00 mg/mL of zinc compared to ddH_2_O-treated controls (Fig. 4B). The mRNA for the *tra* relaxasome (M and I), regulation (J), pilus assembly and extension (E, K, B, P, C, W, and F), pilus maturation (Q and X), transfer (U), type IV coupling protein (D), and surface exclusion (N) all demonstrated significant increases (*P* < 0.05) in expression in conjugations supplemented with zinc gluconate compared to the ddH_2_O-treated controls. The mRNA for the *tra* encoded proteins for pilus assembly (L), extension (V and H), and the multifunctional assembly, stabilization, and inclusion (G) protein demonstrated numerical increases in fold change gene expression but were not significant (*P* > 0.05). The mRNA for the *tra* encoded proteins for regulation (Y and R), and surface exclusion (T) demonstrated no numerical differences in fold change gene expression compared to the water-treated controls (Fig. 4B).

## DISCUSSION

The gut of humans and other animals is a potent reservoir for lateral gene exchange, shedding, and the emergence of novel MDR organisms (8–10, 29–34). Recently, we demonstrated prolific transfer of the MDR plasmid pCVM29188_146 from *Salmonella* Kentucky strain CVM29188 to *E. coli* HS-4 in the gut of a defined microbiota mouse model (17). Additionally, we showed that probiotics combined with a live *Salmonella* vaccine treatment can regulate the transfer of IncF plasmids and a correlation between the concentration of host cecal small RNA and the abundance and distribution of plasmids in gut *Enterobacteriaceae* from chickens (35). Identifying novel and practical approaches to mitigating and regulating this process in the gut is paramount for AMR mitigation and, thus, global health (12, 18).

Little is currently understood about how the host diet and diet-related factors regulate the lateral transfer of bacterial DNA through bacterial plasmid conjugation. Some preliminary studies have shown that components of a regular human diet, such as linoleic acid, may serve as bacterial conjugation inhibitors (12, 18, 19). However, human diets are often supplemented with dietary additives with numerous goals, such as completing daily recommended intake, weight loss, heart function, liver function, and cholesterol management (36–38). Dietary supplements are understudied due to limited regulations; thus, little is known about unintended effects on other aspects of host biology (39). This study aims to elucidate whether dietary zinc supplements may provide some regulatory role in the gut as inhibitors of bacterial plasmid conjugation.

Zinc is a metal cofactor used within cells to mediate enzymatic reactions and must be derived from food or metabolic substrates in the case of microbes (40, 41). Zinc is often supplemented in humans in the form of zinc gluconate, zinc acetate, or zinc sulfate (40). While not the advertised effect of dietary zinc supplements, zinc’s observed antimicrobial impact on bacterial populations was well published (20, 21). Zinc derivatives, such as zinc gluconate and zinc sulfate, are believed to enter the cell and disassociate to form Zn^2+^ ions that contribute to the formation of reactive oxygen species (O_3_, OH, and H_2_O_2_) that can lead to cell killing through cell wall, DNA, and protein damage (22). Interestingly, intracellular damage in bacteria activates the host stress response (SOS) pathway, leading to the global regulation of genes associated with DNA repair, DNA recombination, and metabolism, which was implicated as a stimulatory signal for bacterial plasmid conjugation (42)

Zhang et al. reported the effect of subinhibitory concentrations of heavy metals, including copper [Cu(II)], silver [Ag(I)], chromium [Cr(VI)], and zinc [Zn(II)] on the frequency of conjugation in wastewater environments (42). While they did not report differences in Zn(II)-treated conjugations, they did demonstrate significant induction of conjugation in Cu(II)-, Ag(I)-, and Cr(VI)-treated conjugation reactions compared to untreated controls and attributed these differences to the activation of host SOS response by production of ROS (42). Both Raro et al. and Ekhlas et al. additionally support the observation of zinc derivative stimulation of conjugation through the application of zinc oxide and zinc nitrate as the active zinc molecule, with the latter seeing both stimulatory and inhibitory effects, depending on the strains used (43, 44).

Using supplement-derived zinc gluconate homogenates, we demonstrated a significant bactericidal effect of supplementation on both donor and recipient populations. Additionally, we observed a significant decrease in the total populations of transconjugant bacteria in all three treatment concentrations compared to the control group. While a reduction in total transconjugants is desirable, what is essential in a conjugation inhibitor is the measure of log conjugation efficiency or the ratio at which plasmids are transferred between the viable bacteria in the environment (18, 45). Here, we observed significant reductions in the log conjugation efficiency of all reactions supplemented with supplement-derived zinc gluconate suspensions (Fig. 1D).

It is important to clarify why we observed significant inhibition of plasmid conjugation, while many preliminary studies reported stimulatory activity of heavy metal exposure on plasmid conjugation (42–44). For example, the zinc concentrations reported by Zhang et al. were lesser than those used in this study and may partially explain why they did not observe significant differences in conjugation in zinc-supplemented conjugations. It is also possible that components of the supplement matrix may have contributed to the inhibition of plasmid conjugation observed in our supplement-derived treatment groups.

To evaluate the contribution of extraneous compounds of the zinc gluconate supplement, reagent grade zinc gluconate was obtained; equivalent stock concentrations were prepared; and conjugation assays were repeated. We observed the same trends in total donor, recipient, and transconjugant populations, with increased reductions observed compared to supplement-derived zinc gluconate (Fig. 2). Furthermore, we observed total ablation of transconjugant populations at either 0.5- or 1.0-mg/mL final concentration. These differences in plasmid inhibition observed between supplement- and reagent-derived solutions may be due to variations in the reported vs the actual concentration of active ingredients in dietary supplements, as the supplement used in this study does not report being validated for concentration by a third party (46). To address this, we evaluated the concentration of zinc ions in both solutions using a zinc assay kit (Fig. 3) and found no significant differences between the supplement- and reagent-derived zinc solutions. Alternatively, this variation in efficacy of supplement- and reagent-derived solutions may be due to interference from other materials in the complex supplement matrix, which is noted to include microcrystalline cellulose, dicalcium phosphate, croscarmellose sodium, and less than 2% of carnauba wax, hydroxypropyl methylcellulose, magnesium stearate, natural vanilla flavor, propylene glycol, and stearic acid. It is unclear what effect each specific additive has on plasmid conjugation, or whether or not the additives inhibit the specific mechanisms of zinc gluconate. Additional studies on the individual effects of these additives are required for both evaluations as direct stimulants and inhibitors, as well as to mitigate their inhibition of zinc gluconate COIN activity in the gut during practical application.

Previously, Crane et al. demonstrated the inhibition of AMR gene transfer by the zinc ionophore, zinc pyrithione [bis(2-pyridylthio)zinc 1,1′-dioxide], through the inhibition of the RecA-LexA-mediated SOS response (47). They hypothesized that zinc inhibits the binding of RecA to ssDNA as well as the subsequent RecA-induced cleavage of the LexA SOS repressor (47). However, the contribution of this interaction to plasmid-mediated gene transfer was not investigated. The authors did not characterize the role of RecA and LexA on the processes associated with plasmid conjugation but did report that the genes transferred were potentially located on plasmids (47). Interestingly, the study reported a ~100-fold increase in activity of zinc pyrithione compared to zinc salts in their system, indicating altered activity of this formulation of zinc compared to traditional zinc derivatives associated with dietary supplements. This indicates that these results may not be reflective of what should be expected in supplement-derived zinc interactions with plasmid conjugation.

Next we wanted to clarify why we observed an inhibition of plasmid conjugation under our experimental model while the majority of studies indicate a stimulatory effect of zinc supplementation on conjugation frequency. It is known that induction of the SOS response is associated with an increased expression of the *tra* regulatory protein TraJ that binds to the promoter region of the *traY* gene and results in the increased expression of the *traY* to *traX* polycistronic mRNA (43, 44, 48). However, alterations in this expression may be occurring under the comparably increased concentrations of zinc gluconate used in this study as compared to those in previous studies.

To address this concern, we aimed to evaluate the gene expression of the proteins associated with the plasmid *rep* and *tra* of F-like conjugative bacterial plasmids. We observed a significant upregulation in the expression of the mRNA for the replication initiation protein RepA and a numerical increase in the expression of RepB, both located disparately in the plasmid genome and regulated by separate elements. RepA is a replication initiator protein that is required for the replication of plasmids of IncF incompatibility group (49, 50). The binding of monomeric RepA to iteron repeat sequences (RepA binding motifs) near the origin of replication is essential for plasmid replication and is implicated as a potential Mob alternative to assist in plasmid conjugation (51, 52). RepA binding is rate limiting and regulates plasmid copy number in iteron-regulated plasmid types, such as IncFIβ (51). However, over-expression of the RepA protein is postulated to result in inhibitory effects on plasmid replication through a dimeric conformation that leads to handcuffing inhibition of plasmid replication (51). Reductions in plasmid replication due to RepA dimer binding may partially explain why we observe reductions in plasmid conjugation in reactions supplemented with zinc gluconate and are the subject of ongoing studies.

In addition to the alteration of replication-associated genes, a significant increase in expression of the majority of *tra* genes was observed with the exception of the relaxasome regulatory gene *traY*; the pilus assembly genes *traL*, *traV*, and *traH*; the polymerase-binding transcriptional regulator gene *traR*; the coupling/ATPase gene *traD*; the pilus assembly, mating pair stabilization, and exclusion gene *traG*, and the surface exclusion gene *traT* (6). Alternatively, expression of the *traA* and *traS* genes was observed to have significant decreases in log fold change in gene expression (Fig. 4) (51).

The *traA* gene encodes the conjugal propilin polypeptide protein that, after modification, is essential to producing the f-like type IV secretion system conjugation machinery (50). The TraQ chaperon protein traffics the propilin to the inner membrane, where the signal peptide sequence is inserted into the inner membrane and is cleaved by host LepB protease to form the mature TraA pilin protein and a residual tail signal peptide (50). The mature TraA pilin peptide is then believed to be n-acetylated by the TraX protein before being incorporated into the pilus assembly apparatus or stored in the inner membrane in pools for rapid assembly and disassembly (50, 53). Manchak et al. reported over-expression of non-acetylated pilin does not affect the formation of conjugal pili; however, formal under-expression of mature pilin has not yet been fully characterized (54). It is understood, however, that the number of cell surface conjugal pili is limited by the expression level of pilin, with increased expression corresponding to increases in the number of conjugal pili on the cell surface (7, 54). In mutational knockdown or inhibition studies, alterations in expression level or modifications to the amino acid sequence have been linked to partial or complete inhibition of conjugation frequency (54). While the contribution of decreased intracellular pools of *traA* to the inhibition of plasmid conjugation appears obvious, the specific mechanism of TraA protein concentration effect on conjugation is still not clear and is currently under further evaluation.

The *traS* gene encodes the entry exclusion protein TraS, the expression of which is critically important in recipient cells already containing F-like plasmids and prevents the fertile transfer of plasmids between donors and existing transconjugants (53, 55, 56). The TraS protein acts in transconjugants to prevent lethal zygosis and the disruption of the structural cell membrane due to the multiplicity of conjugations and cell stress (7, 56). The decrease in expression of *traS* gene mRNA observed in conjugations supplemented with zinc gluconate may indicate that either the donor cells, the recipient cells, or the combination of both is under-expressing the proteins required for entry exclusion, resulting in potentially significant host cell lethality. The combination of excessive conjugation stress with the expected increase in cellular ROS concentrations associated with exposure of *E. coli* to zinc gluconate may explain the significant donor and recipient morbidity as well as reductions in total transconjugant populations and conjugation efficiency. The specific role of *traS* gene expression modulation in zinc gluconate inhibition of plasmid conjugation must be further evaluated.

Currently, expression of most *tra* genes is believed to occur under the conserved regulation of the *traY* promoter as a polycistronic mRNA with the exception of the genes *traM* and *traJ*, both of which have known independent regulatory promoter sequences (6, 50, 57, 58). However, how supplement-derived zinc gluconate directs the under-expression of *traA* and *traS* transcripts while maintaining increased expression of many other genes remains unclear and may be attributable to post-transcriptional modification or inhibition of detection via RT-qPCR due to the presence of reverse transcription inhibitors such as antisense RNA (59, 60). The evaluation of differential mRNA expression of *tra* genes assumed to be associated with the *traY* polycistronic mRNA is ongoing. Furthermore, these data indicate there is an inhibitory effect on the model conjugal pair used in this study. Further evaluation is required to determine if zinc inhibition is universal across host strain/plasmid combinations, and this work is ongoing.

Additionally, although the use of the 16S rRNA mRNA as an internal control for qPCR is debated [reviewed in reference (61)], it is still currently used as the reference gene in the majority of qPCR methods for quantification of the change in gene transcript expression in bacterial conjugation assays (62–67). However, if variation in 16S rRNA gene expression was a source of error, we would not expect to observe the combination of over-, normal-, and under-expression in *tra* genes as we did and would likely see global upregulation or downregulation of all genes assayed under conditions of altered 16S rRNA gene expression. Future identification of alternative housekeeping genes that are validated for stable expression under bactericidal or inhibitory conditions is desired and ongoing. Furthermore, for the RT-qPCR studies, perfect controls for donor and or recipient gene expression in the absence of the cognizant mating pair strain are not immediately possible due to the influence of matting pair formation on the expression of conjugation genes (49, 50, 57, 68, 69). Further work is required to identify methods that will aid in the identification of source gene expressions in order to identify the differential expression of conjugation genes within each member of the conjugation reaction.

Overall, this study identifies a potential role of supplement- and reagent-derived zinc gluconate as a source of biologically active zinc that demonstrates both a bactericidal effect on donors and recipients and a role as a plasmid COIN *in vitro*. Inhibition is further correlated with shifts in the expression of *rep* and *tra* genes critical for fertile plasmid conjugation (50, 53). These data indicate that supplement-derived zinc gluconate may serve as a potential intervention for mitigating the emergence and spread of novel plasmid-associated MDR bacteria in the gut. Further studies are required on the efficacy and application of zinc gluconate supplementation on the reduction of conjugation in the complex gut environment, as well as to elucidate the full mechanism of inhibition.

### Conclusion

Herein, we identified a potential role for supplement-derived zinc as a regulator of bacterial populations and bacterial plasmid conjugation *in vitro*. While the complete mechanism behind this interaction is not yet apparent, we have evidence to support that conjugation inhibition is driven by a differential shift in gene expression associated with plasmid replication and transfer genes. Diet-associated supplements may play a significant role in the regulation of the incidence and abundance of bacterial plasmid conjugation in the human and animal gut and need to be further explored to determine the significance and practicality of using this approach to mitigate the global epidemic of MDR emergence.
